# Supplementary material for: Pupil Constriction Causes Activity in the Human Retina and Visual System
Source: Psychophysiology. 2026 Jul 22;63(7):e70362. doi: 10.1111/psyp.70362 (PMC13392059; doi:10.1111/psyp.70362)
Supplement: Supplementary file 1 — Figure S1: (a) Stimulus‐locked ERG separately for upper and lower electrodes. (b) Constriction‐locked ERG separately for upper and lower electrodes. Figure S2: As main Figure 2, but after removing all independent components that were not labeled brain or other. Figure S3: The ERG signal locked to stimulus onset after 5 Hz high‐pass filtering for all trials, sorted by timepoint of maximum constriction velocity. Figure S4: As main Figure 2, but based on a recovery analysis with simulated data. [file PSYP-63-e70362-s001.docx]

Supplementary Materials for

Pupil Constriction Causes Activity in the Human Retina and Visual System

Sebastiaan Mathôt [1], Olaf Dimigen [1], Hakan Karsilar [1], Veera Ruuskanen [1],

Daria Weiden [2], and Ana Vilotijević [1]

1. Department of Psychology, University of Groningen, The Netherlands

2. Faculty of Science and Engineering, University of Groningen, The Netherlands

Address for correspondence:

Sebastiaan Mathôt

Department of Experimental Psychology

University of Groningen

Grote Kruisstraat 2/1

9712TS Groningen

The Netherlands

Email: s.mathot@rug.nl

# Supplementary Materials

## Datasets

All datasets were collected in the same laboratory under similar conditions. However, the task and stimulus parameters differed as described in the table below. Some datasets were collected in a single experimental session with the same participants (e.g. Follow-up Bright and Follow-up Dark).

| Dataset | N_subjects_ | N_trials_ | EEG | Stimulus duration(s)  (ms) | Stimulus luminance(s)  (cd/m^2^) | Background  display  luminance (cd/m^2^) | Environment illuminance (lux) | Task |
| --- | --- | --- | --- | --- | --- | --- | --- | --- |
| Original  [(Mathôt et al., 2024)](https://www.zotero.org/google-docs/?4jxNgq) | 10 | 3,865 | Yes | 100 | 2.69, 9.84, 24.24, 48.45, 85 | < .01 | < 1 | To detect a rare target (10% present) embedded in the stimulus. Target-present trials excluded from analysis. |
| Follow-up Bright | 22 | 4,889 | Yes |  | 48.3, 84.8 |  | 743 |  |
| Follow-up Dim |  | 4,540 |  |  |  |  | < 1 |  |
| Timing | 27 | 3,474 | Yes | 200, 300, 400, 500, 600, 700 | 2.55, 5.75, 11.55, 21.63, 41.24, 77.66 |  |  | To indicate stimulus duration. |
| Brightness |  | 3,152 |  |  |  |  |  | To indicate stimulus brightness. |
| Attention 1 Bright [(Vilotijevic, 2026)](https://www.zotero.org/google-docs/?6X3Vd9) | 28 | 6,370 | No | 100 | White noise with (opacity: 10, 40, 100%) presented on bright side. | Display divided into a bright (99.60) and dim (0.14) half. |  | To indicate the position (above or below midline) of a small circular target embedded in the stimulus. |
| Attention 1 Dim  [(Vilotijevic, 2026)](https://www.zotero.org/google-docs/?5pRlEL) |  | 6,147 |  |  | White noise with (opacity: 10, 40, 100%) presented on dim side. |  |  |  |
| Attention 2 Bright  [(Vilotijevic, 2026)](https://www.zotero.org/google-docs/?YOJEod) | 32 | 8,352 | Only occipital electrodes |  | 0.14 | 99.60 | 743 | To detect a target letter (F; 50% present) surrounded by flankers (E’s) embedded in the stimulus. |
| Attention 2 Dim  [(Vilotijevic, 2026)](https://www.zotero.org/google-docs/?UG4h64) |  | 7,720 |  |  | 99.60 | 0.14 | < 1 |  |
| Total | 119 | 48,509 |  | | | | | |

##

## The constriction-locked ERG component does not result from blinks or eye movements

Signals that come from the retina affect all EOG electrodes similarly. For example, the a- and b-wave (but not later parts of the signal) are identical for upper and lower electrodes (Sup. Fig. 1). In contrast, signals that result from blinks and eye movements tend to have opposite-polarity effects on upper and lower EOG electrodes. Therefore, to exclude the possibility that the constriction-locked ERG component is in fact caused by blinks or eye movements, we visualized the upper and lower electrodes separately. Although the constriction-locked ERG component is somewhat stronger for the upper electrodes, the polarity (a trough followed by a peak) is the same for both upper and lower electrodes, suggesting a retinal origin.


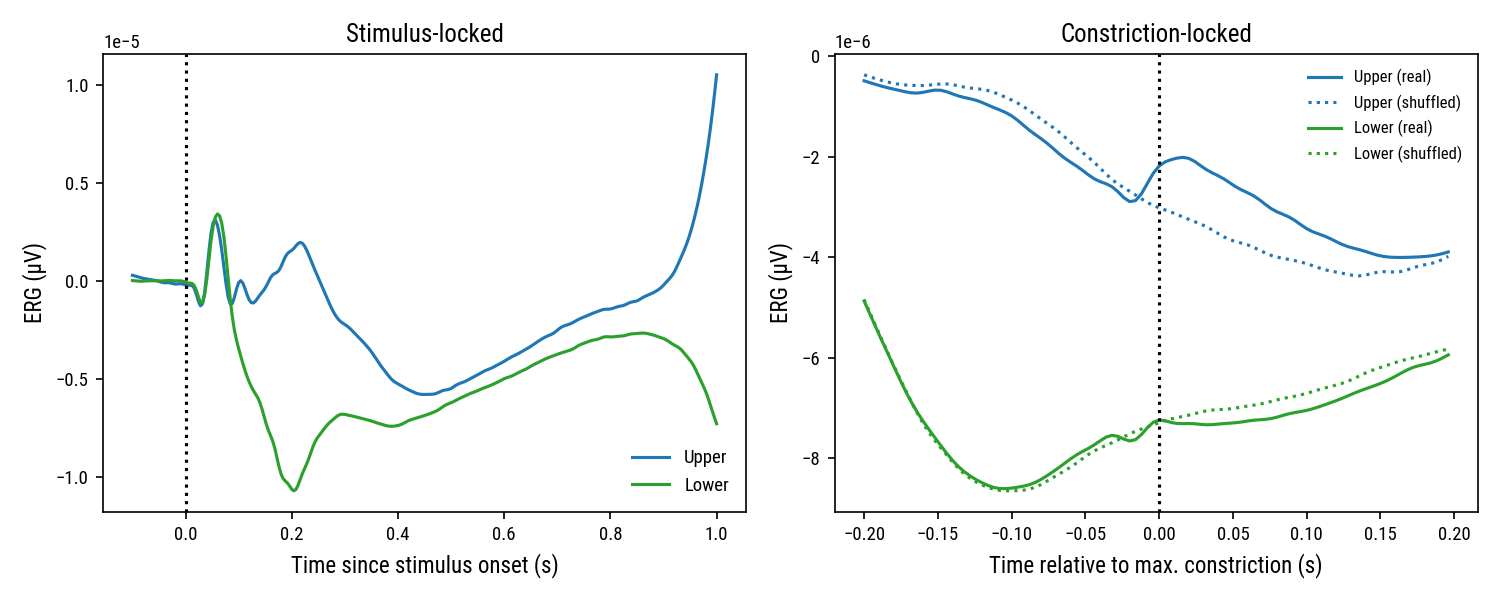


##### **Supplementary Figure 1.** a) Stimulus-locked ERG separately for upper and lower electrodes. b) Constriction-locked ERG separately for upper and lower electrodes.

In a separate, complementary control analysis to exclude contributions of blinks and eye movements, we repeated the entire analysis after removing independent components from the signal during preprocessing. Specifically, we used the ICA-label library [(Li et al., 2022)](https://www.zotero.org/google-docs/?lkTeDJ) to automatically classify independent components as reflecting one of the following sources: brain, muscle artifact, eye blink, heart beat, line noise, channel noise, and other. We removed all components except those labelled as brain or other. Re-assuringly, this did not notably affect the constriction-locked ERG component. (For the main analysis, we did not remove any independent components, because we are not entirely sure how this may affect signals from the retina, although based on this control analysis the impact seems small.)


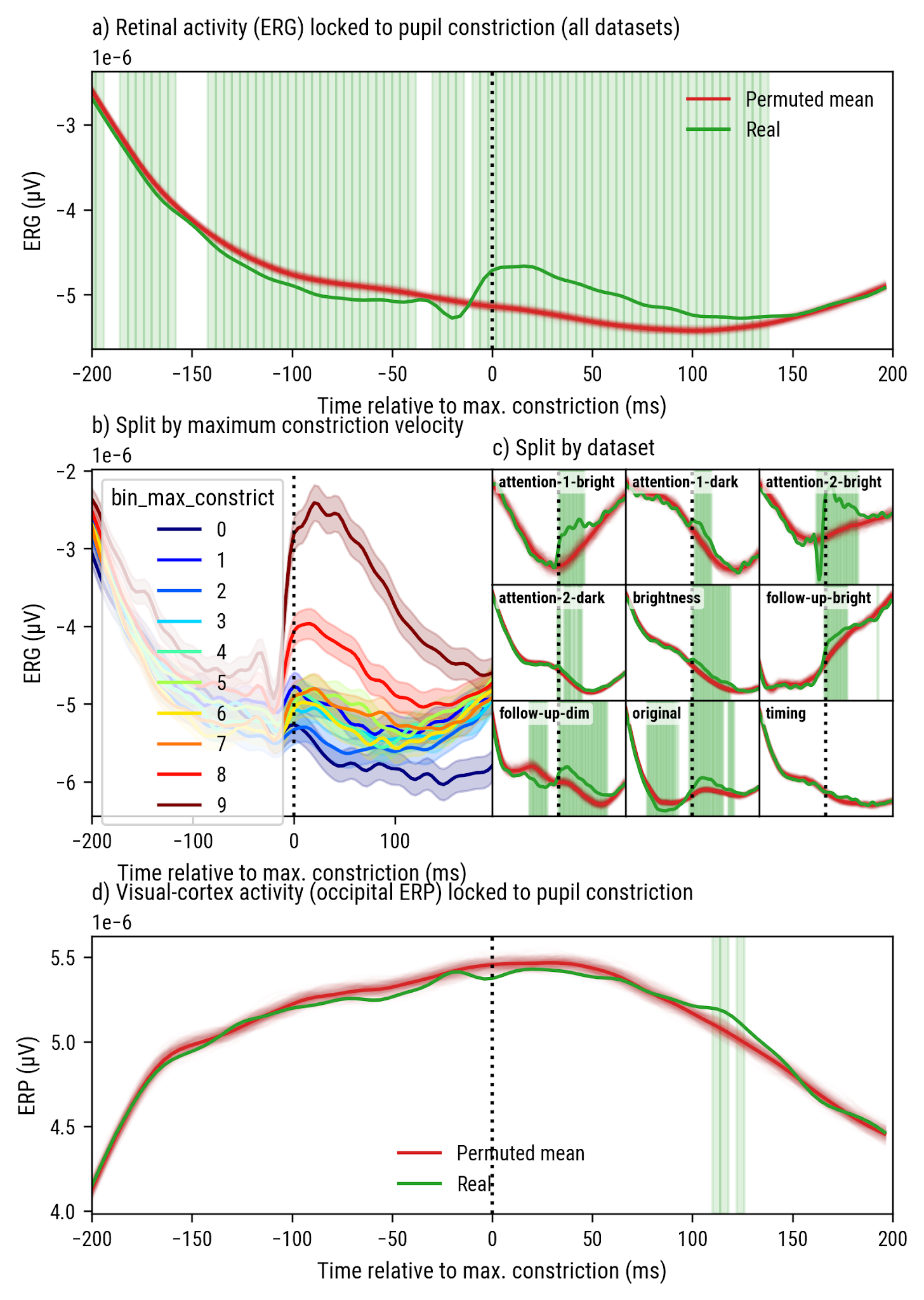


##### **Supplementary Figure 2.** As Main Fig. 2, but after removing all independent components that were not labelled brain or other.

## The constriction-locked ERG component is (weakly) visible in a trial-sorted plot

The figure below plots the 5Hz high-pass filtered ERG signal over time for all trials in all datasets. The a-wave and b-wave are clearly visible as vertically running peaks (yellow) and troughs (blue) following stimulus onset. Trials are sorted based on the timepoint of maximum constriction, annotated in red. The constriction-locked ERG component, which overall is about 10 times smaller in amplitude than the a-wave and b-wave, is weakly visible, mainly at the bottom of the plot.


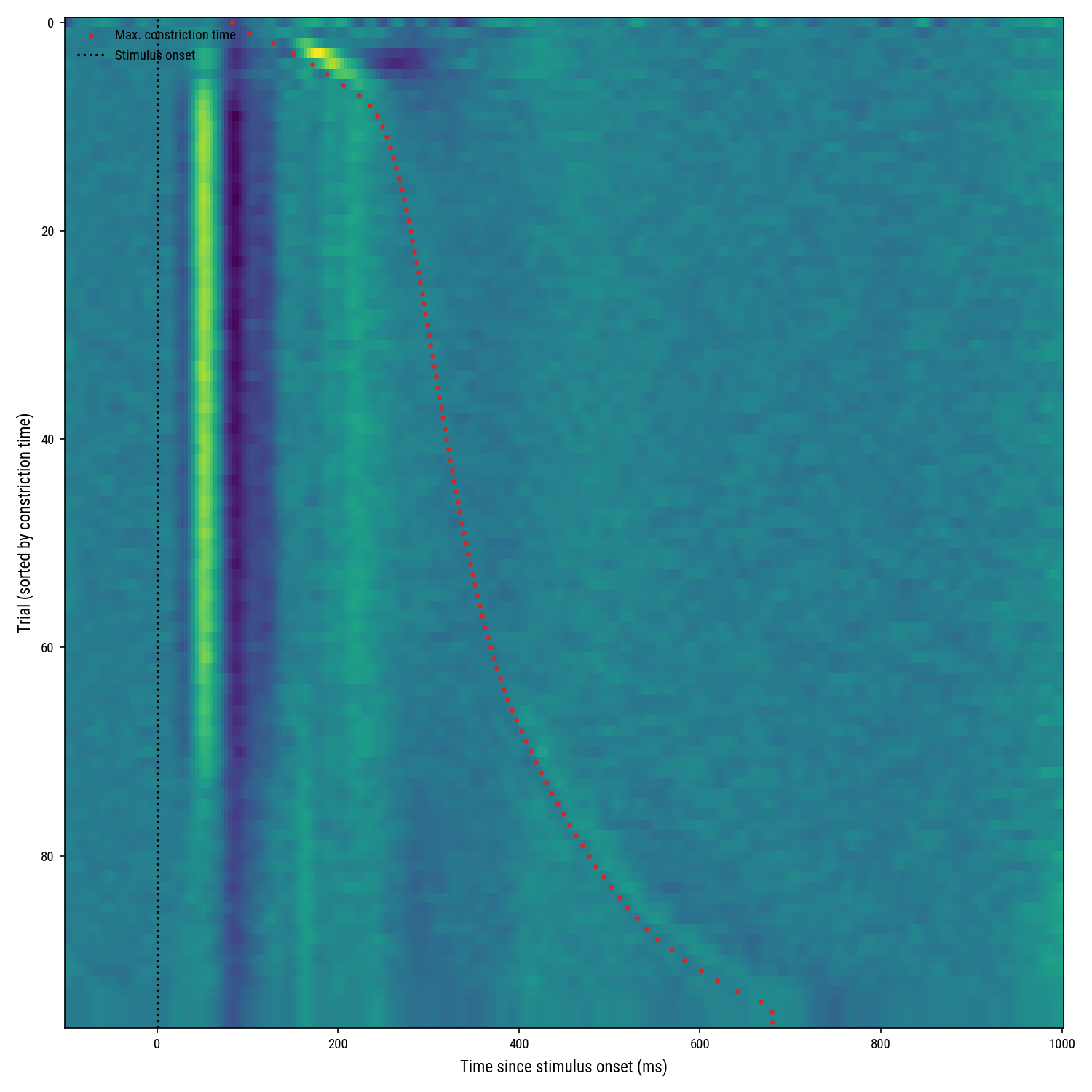


##### **Supplementary Figure 3.** The ERG signal locked to stimulus onset after 5Hz high-pass filtering for all trials, sorted by timepoint of maximum constriction velocity.

## A recovery analysis with simulated data shows the validity of the analysis pipeline

We were initially struck that such a clear ERG response emerged when locking the signal to the timepoint of maximum constriction velocity, given that the trial-to-trial variability in constriction latency is modest. We therefore ran a control analysis with simulated data to verify that this is indeed possible. Specifically, we replaced the real ERG signal with one of the permuted baselines, and added a single sine wave cycle around the timepoint of maximum constriction velocity with an amplitude comparable to the constriction-locked ERG response (0.5µV). We then repeated the entire analysis. As shown in Sup. Fig. 4, the simulated constriction-locked ERG is successfully recovered.

##### **
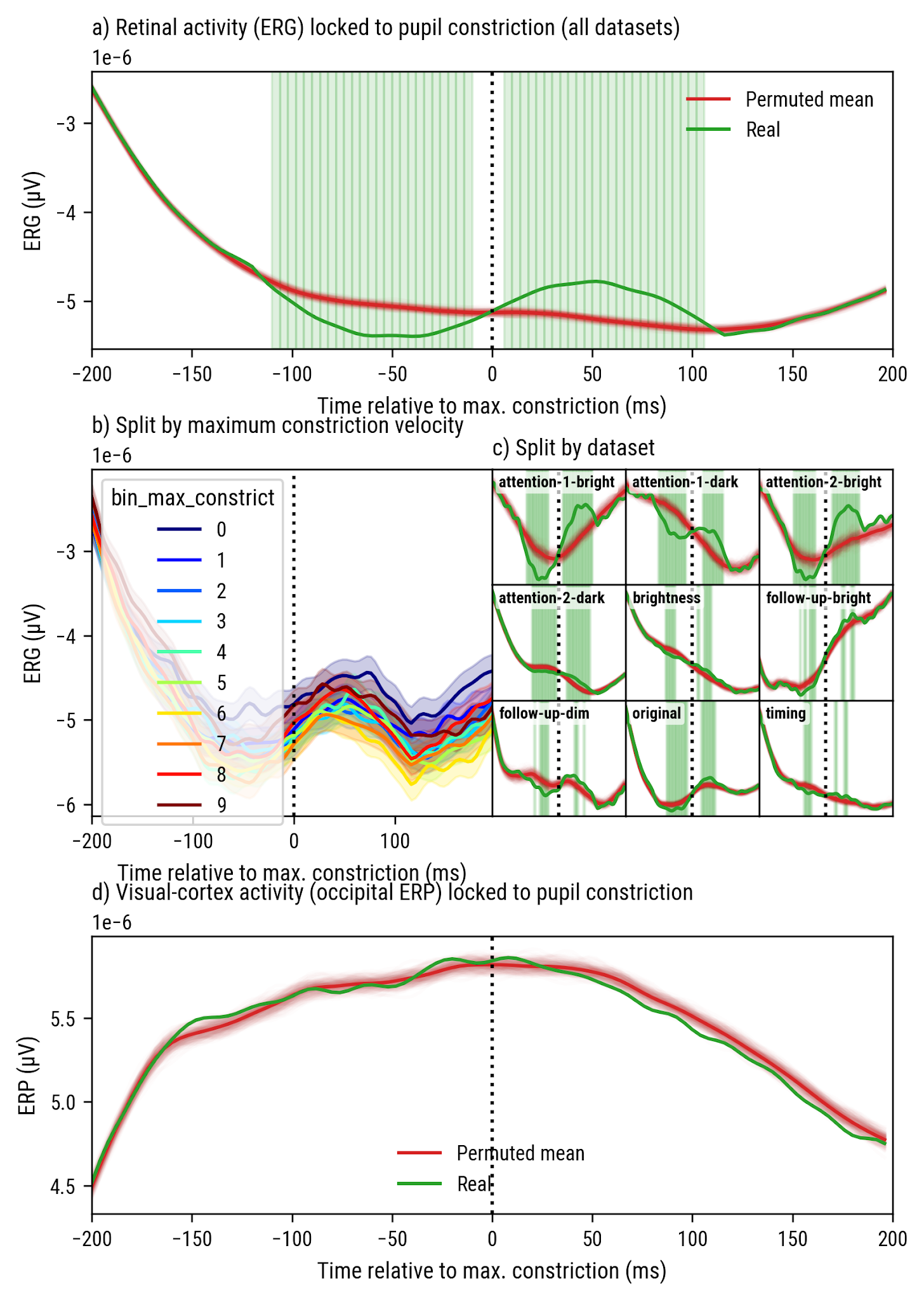
**

##### **Supplementary Figure 4.** As Main Fig 2, but based on a recovery analysis with simulated data.
